# Foreign Summary

**Published:** 1838-04-11

**Authors:** 


### FOREIGN SUMMARY.

An hospital has been lately established at Canton, by an American Surgeon and Missionary, the Rev. P. Parker, for the gratuitous relief of afflicted Chinese. Several important operations have been performed, which have created considerable excitement among the Chinese, whose ignorance of surgery and medicine is said to be extreme. Such an institution may lead to the opening of an extended intercourse with this singular people, of whom we as yet know so little.

---

M. Amussat, in a paper in the Memoirs of the Royal Academy of Medeicine, considers the twisting of arteries, or the method of torsion, fully as effectual in stopping hemorrhages as the ligature; while it is often more ready in its application, as it only requires the aid of the operator alone and a pair of pincers, instead of the addition of an able assistant, who is not always to be met with in sudden emergencies. M. Amussat represents this method of securing arteries as advancing, though slowly, and as steadily recommending itself among operating surgeons in France, notwithstanding the opposition with which it might naturally be supposed that it would have to contend.—*Edin. Med. and Surg. Journal.*

---

*Hydro-sulphuretted Baths.*—The beneficial effects of sulphur, in diseases of the skin, are well known to all practitioners. It has also been established, that, when administered in baths, sulphur acts more powerfully than when given under any other form. M. Montain, professor at the *School of Medicine*, of Lyons, has lately composed a substance which is well calculated for the preparation of these baths. The following is the formula which he employs.

*Powdered sulphuret of calcium*, 8 parts.

*Hydrochlorate of soda*, 2 parts; Mix with one part of any cheap vegetable extract.

*Isinglass*, 1 part. Make up the whole into balls containing about an ounce and a half.

This preparation possesses the advantages of being very cheap, of not emitting any disagreeable odour, or acting on the vessel which may contain the water.—*London Lancet.*

---

*History of a Female who has four Mammæ and Nipples. By* Robt. Lee, M. D., F. R. S., Physician to the British Lying-in Hospital, and Lecturer on Midwifery at St. George's Hospital.

The individual in whom the above-mentioned peculiarity presented itself was thirty-five years of age, and was prematurely delivered of a still-born child, on the 20th of July, 1835. The mammæ having afterwards become excessively painful and distended, she was compelled to permit the author to make an examination of them, by which it was discovered that she had two mammæ and two nipples on each side. The inferior or pectoral mammæ were fully developed, and in the natural situation, and their nipples, areolæ, and glands presented nothing unusual in their appearance. Near the anterior margins of the axilla, a little higher up on each side, was situated another mamma, about one-sixth the size of the others. The nipples of these were small and flat, but when gently pressed, a milky fluid flowed copiously from several ducts which opened upon their extremities. When milk was drawn from the lower breasts, a small quantity usually escaped from the nipples of the upper, and when the draught came into the former, the latter invariably became hard and distended. From the flatness of the nipples of the upper breasts, the patient had never been able to suckle with them.—*London Lancet.*

